# Mobility and social identity in the Mid Upper Paleolithic: New personal ornaments from Poiana Cireșului (Piatra Neamț, Romania)

**DOI:** 10.1371/journal.pone.0214932

**Published:** 2019-04-24

**Authors:** Elena-Cristina Nițu, Marin Cârciumaru, Adrian Nicolae, Ovidiu Cîrstina, Florin Ionuț Lupu, Marian Leu

**Affiliations:** 1 “Princely Court” National Museum Târgovişte, Museum of Human Evolution and Technology in Paleolithic, Târgovişte, Dâmboviţa County, Romania; 2 Valahia University of Târgovişte, Doctoral School, Târgovişte, Dâmboviţa County, Romania; Universita degli Studi di Ferrara, ITALY

## Abstract

Most of the Paleolithic art and ornaments discovered in Romania come from the site of Poiana Cireșului. Four Paleolithic layers have been studied at this site—the oldest one belongs to the Early Gravettian period between 30 ka and 31 ka BP. The ornaments discovered in this layer include perforated shells from three species of mollusks: freshwater *Lithoglyphus naticoide* and *Lithoglyphus apertus* as well as *Homalopoma sanguineum* (an exclusively Mediterranean species). Poiana Cireșului is one of the very few Gravettian sites where perforated *Homalopoma sanguineum* shells were found, and the importance of this discovery is stressed even more by the very long distance between the site and the nearest source located over 900 km away. This find suggests the connection of communities here with the Mediterranean area as well as a possible movement of populations from the south of the continent to the east of the Carpathians with significant implications in understanding human group mobility and the origin of the Early Gravettian in this area. Furthermore, Poiana Cireșului is the only Gravettian settlement where *Lithoglyphus naticoides* shells were used. The unique association of perforated shells—not found in any other Gravettian settlement—contributes to the identity of the Paleolithic community of Poiana Cireșului through their ornaments.

## Introduction

Gravettian settlements are concentrated in two areas in Eastern Europe: The Russian Plain, particularly the Kostenki-Borchevo region, and east of the Carpathians along the valleys of the Bistrița, Prut and Dniester rivers. The origin and diffusion of the Gravettian remain under debate [1, 2] alongside the issues of unity or diversity within this culture [3, 4, 5]. Whether characterized by reference to the evolutionary stages of the Central and Eastern European Gravettian [6, 7, 8] or included in a local culture called the *Molodovian* [9], the analysis of settlements to the east of the Carpathians has not yet provided a coherent picture of the cultural dynamics of the Gravettian in this area. More than 20 sites with Paleolithic finds have been identified in the Bistrița valley [10, 11], but only Poiana Cireșului has been subjected to systematic archaeological research. Unlike other settlements, it preserves a long chronostratigraphic succession and various traces of material culture (faunal remains, lithic and bone tools and weapons, art objects and adornments, hearths and dwelling traces) as well as the oldest Gravettian layer in this area dated around 30–31 ka cal. BP. [12, 13]. Moreover, it is the only site in the Bistrița valley where ornaments have been discovered [14].

The recent discovery of a unique assemblage of ornaments in the oldest Gravettian occupation of Poiana Cireșului contributes substantially to the characterization of the Mid Upper Paleolithic east of the Carpathians and questions the previous theories on the central European or local origin of the Gravettian. The ornaments feature perforated shells of freshwater and marine gastropods (Mediterranean origin) belonging to species that are very rare in the Gravettian: *Lithoglyphus naticoides*, *L*. *apertus* and *Homalopoma sanguineum*. The discovery of the perforated *Homalopoma sanguineum* shells, an exclusively Mediterranean species, has many implications regarding the identity of Gravettian communities east of the Carpathians, the establishment of movement directions, the mobility and connections among groups. The ornaments and other features of occupation in this site highlight the great differences versus the Gravettian settlements in Central and Eastern Europe. In this study, we analyzed the perforated shells discovered and discuss the implications of the particular association of Poiana Cireșului ornaments in understanding the Gravettian.

## Materials

**Fig 1:**
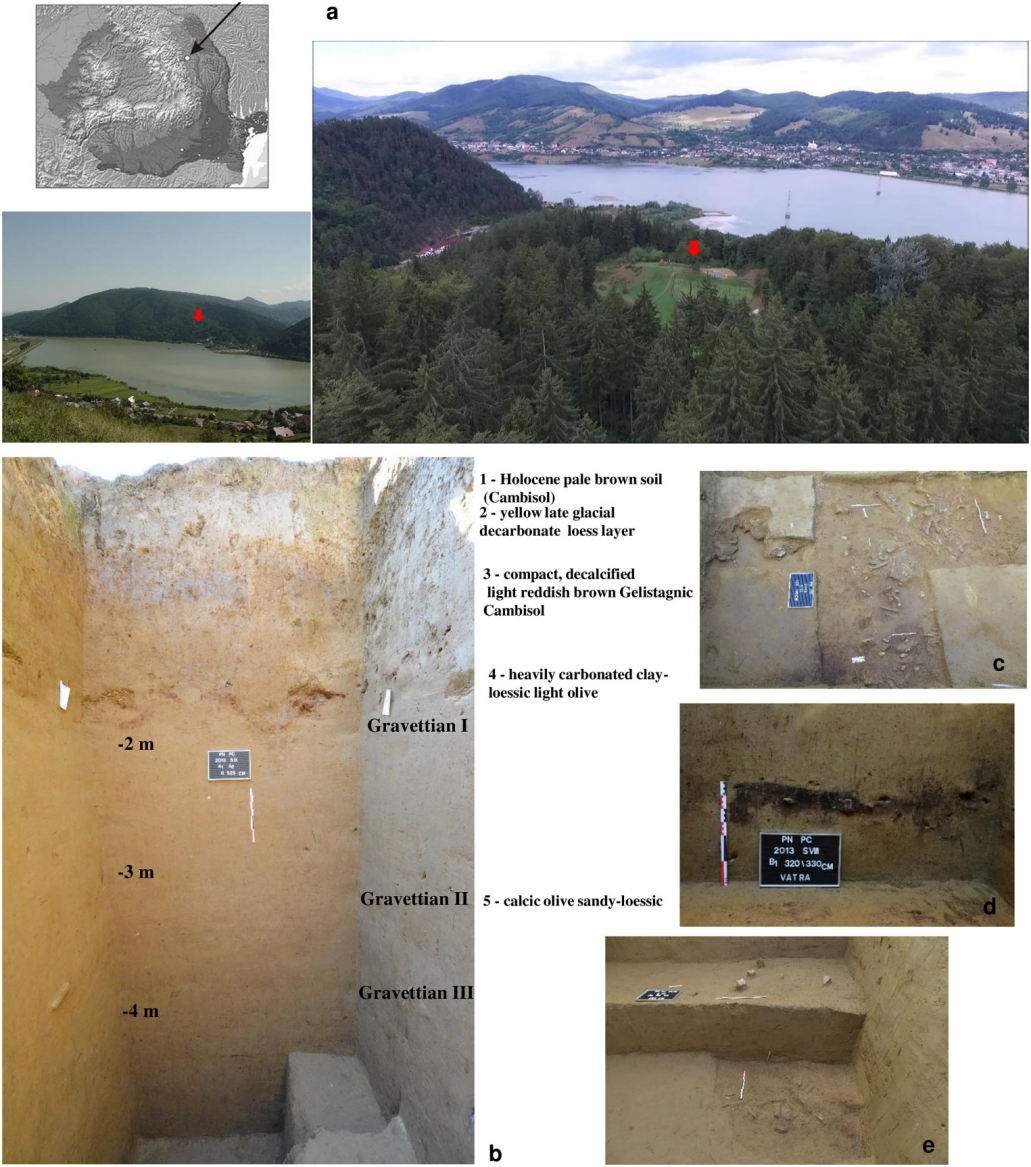
The settlement of Poiana Cireșului. (a) Site location. (b) Stratigraphic profile in section IX and the sequence of Gravettian layers. (c) Image during excavation, Gravettian I. (d) Hearth discovered in section VIII, Gravettian II. (e) Stratigraphic sequence of Gravettian II and III in section XII.

The Paleolithic settlement of Poiana Cireșului-Piatra Neamț (Neamț County) is located on cut terraces of the Bistrița river on the right bank at the confluence with the Doamna rivulet (46°55'919'' North latitude and 26°19'644" East longitude), 395 m absolute altitude (Fig 1). Systematic archaeological excavations underway since 1998 have uncovered an area of about 100 m^2^ including four Paleolithic layers [12, 13, 14, 15, 16] (S1 Fig). Surveys have revealed that the geological deposit is 8 m thick in some areas. There are traces of possible earlier layers detected under the oldest Gravettian occupation. In terms of geological stratigraphy, the following sequence has been identified: 1—Holocene pale brown soil (cambisol); 2—yellow late glacial decarbonated loess layer; 3—compact, decalcified light reddish brown gelistagnic cambisol; 4—heavily carbonated clay-loessic light olive layer; and 5—calcic olive sandy-loessic layer.

The four Paleolithic occupational levels investigated here are separated by thick sterile layers with the following cultural sequence (Fig 1b):

- Layer I is located in the second geological deposit near the soil surface (Fig 1b). It has only lithic materials and is partially disturbed in places by a Neolithic occupation; this level has been attributed to the Epigravettian based on the stratigraphic position and the features of the lithic material.
- Layer II is between 170–210 cm in the fourth geological level (Fig 1b and 1c). It is dated between 19,320 ± 80 uncal. BP (OxA-36785) (22–23 ka cal. BP) and 20,154 ± 97 uncal. B.P. (ER 12.163) (23–24 ka cal. BP) (Table 1). It is the richest cultural layer at Poiana Cireșului with more than 15,000 lithic items, more than 20,000 osteological remains, an organic material industry and portable art objects [14, 17, 18]; it has been attributed to the Late Gravettian (hereinafter Gravettian I). This layer contains several occupational sequences that are difficult to separate in the excavation because of the extremely high density of materials.
- Layer III (hereinafter Gravettian II) is located between 290 and 310 cm (the contact between geological deposits 4 and 5) (Fig 1b, 1d and 1e). It dates to around 24,500 uncal. BP. (28–29 ka cal. BP) (Table 1). Layer III has several hundred lithic items and osteological fragments; it is probably defined by short occupational sequences.
- Layer IV (hereinafter Gravettian III) is located between 375 and 415 cm (fifth geological deposit) (Fig 1b and 1e) with an age near 26,000 uncal. BP (30–31 ka cal BP) (Table 1). It contained over 3,000 lithic items and osteological remains belonging to large herbivores (Bos/Bison), very thick hearths as well as traces of dwelling structures and pits. The stratigraphy contains two occupational sequences that cannot be traced over the entire area; it is the earliest Paleolithic occupation in the Bistrița valley.

**Table 1:** Absolute dating for Gravettian layers at Poiana Cireșului-Piatra Neamț site.

Depth (cm)	Layer	Material	AMS Lab. Nr.	Age BP (uncal.)	Age (cal. BP) 95.4% probability, for calibration, see details in [19]
185	II (Gravettian I)	Charcoal	RoAMS 63.33	16,850±63	20,529–20,100
185	II	Bone	RoAMS 67.33	18,607±87	22,696–22,300
183	II	Tooth	RoAMS 68.33	18,819±96	22,941–22,450
183	II	Charcoal	OxA-36785	19,320±80	23,538–22,992
190	II	Charcoal	ER 12.162	19,459 ± 96	23,730–23,085
185	II	Tooth	OxA-X-2762-24	19,440±130	23,779–23,020
182	II	Charcoal	OxA-36786	19,555±80	23,856–23,265
185	II	Charcoal	RoAMS 65.33	19,571±67	23,850–23,320
184	II	Charcoal	RoAMS 64.33	19,615±105	23,961–23,321
184	II	Tooth	RoAMS 69.33	19,640±87	23,948–23,390
190	II	Charcoal	RoAMS 62.33	19,710±64	23,981–23,506
180	II	Reindeer tooth	OxA-X-2762-23	19,790±180	24,277–23,385
185	II	Charcoal	RoAMS 66.33	19,836±83	24,125–23,610
186	II	Tooth	RoAMS 71.33	19,881±91	24,195–23,645
192–193	II	Charcoal	Beta 224.156	20,020±110	24,380–23,790
210	II	Charcoal	Beta 244.071	20,050±110	24,408–23,828
207	II	Charcoal	ER 9.964	20,053±88	24,370–23,869
210	II	Charcoal	ER 9.965	20,076±185	24,601–23,648
210	II	Charcoal	ER 12.163	20,154±97	24,484–23,959
303	III (Gravettian II)	Bone (bos/bison)	OxA-X-2762-25	23,420±310	28,200–27,112
295	III	Charcoal	OxA-36787	23,820±110	28,128–27,656
318	III	Charcoal	RoAMS 60.33	24,410±127	28,753–28,511
330	III	Charcoal	OxA-36788	24,540±120	28,854–28,283
320	III	Charcoal	RoAMS 61.33	24,566±88	28,827–28,369
318	III	Charcoal	OxA-36789	24,820±120	29,187–28,553
303	III	Charcoal	Beta 244.072	25,135±150	29,556–28,801
339	IV (Gravettian III)	Charcoal	OxA-36790	25,390±140	29,895–29,030
350	IV	Charcoal	OxA-36768	25,460±200	30,232–29,033
365	IV	Charcoal	OxA-36792	25,650±150	30,340–29,380
364	IV	Charcoal	Beta 244.073	25,760±160	30,491–29,476
382	IV	Charcoal	Beta 224.157	25,860±170	30,620–29,570
371	IV	Charcoal	Beta 206.707	26,070±340	30,943–29,519
408	IV	Charcoal	ER 9.963	26,185±379	31,038–29,553
365	IV	Charcoal	OxA-36791	26,250±140	30,920–30,197
415	IV	Charcoal	ER 9.962	26,347±387	31,140–29,676
360	IV	Bone (bos/bison)	OxA-36914	26,480±230	31,100–30,271
360	IV	Bone (bos/bison)	OxA-36915	26,610±230	31,158–30,440
375–415	IV	Charcoal	ER 11.860	26,677±244	31,204–30,487
375–415	IV	Charcoal	ER 11.859	27,321±234	31,551–30,921

The samples marked as OxA-X had very low collagen yield (<1% weight collagen). All these dates were calibrated with Oxcal computer program (v4.3), using the ‘IntCal13’ dataset.

All the perforated shells analyzed in this article have been discovered in the oldest occupation excavated at Poiana Cireșului, i.e., the Gravettian III. Most of the perforated shells were collected in only two areas of the site (sections V and XII) where traces of dwellings and hearths have been identified (S1 Fig). The state of preservation of the materials in this layer is rather good despite the deposits of carbonates that are often found on items particularly on bones.

The lithic material has been partially analyzed and published [12, 13, 20] and is still under study. Our observations of the Gravettian III focus on materials found in the 2016 and 2017 campaigns. The items are made mostly of flint and siliceous sandstone with less menilite, chert, and black schist. The siliceous sandstone, menilite and cherts are all local rocks. The flint used in this layer has been defined as a rock that was brought from the Prut valley about 200 km away [13]. The tools are almost exclusively made of flint with only a few retouched items of siliceous sandstone. The typological range is very narrow and is defined mainly by backed tools (backed bladelets, microgravettes, Gravette points) as well as a few retouched flakes and blades, some endscrapers on thick and large blades, a tanged tool as well as a few splintered pieces. Burins are absent, although they were counted as rare in previous analyses [20] and should be re-examined for better identification.

From our observations, the attribution of this layer to the Gravettian grounds on the presence of backed tools. Except for the perforated shells, no pendants made of perforated teeth, which are very common in the Gravettian, no decorated objects and no tools or points of bone, antler or ivory have been found.

**Fig 2:**
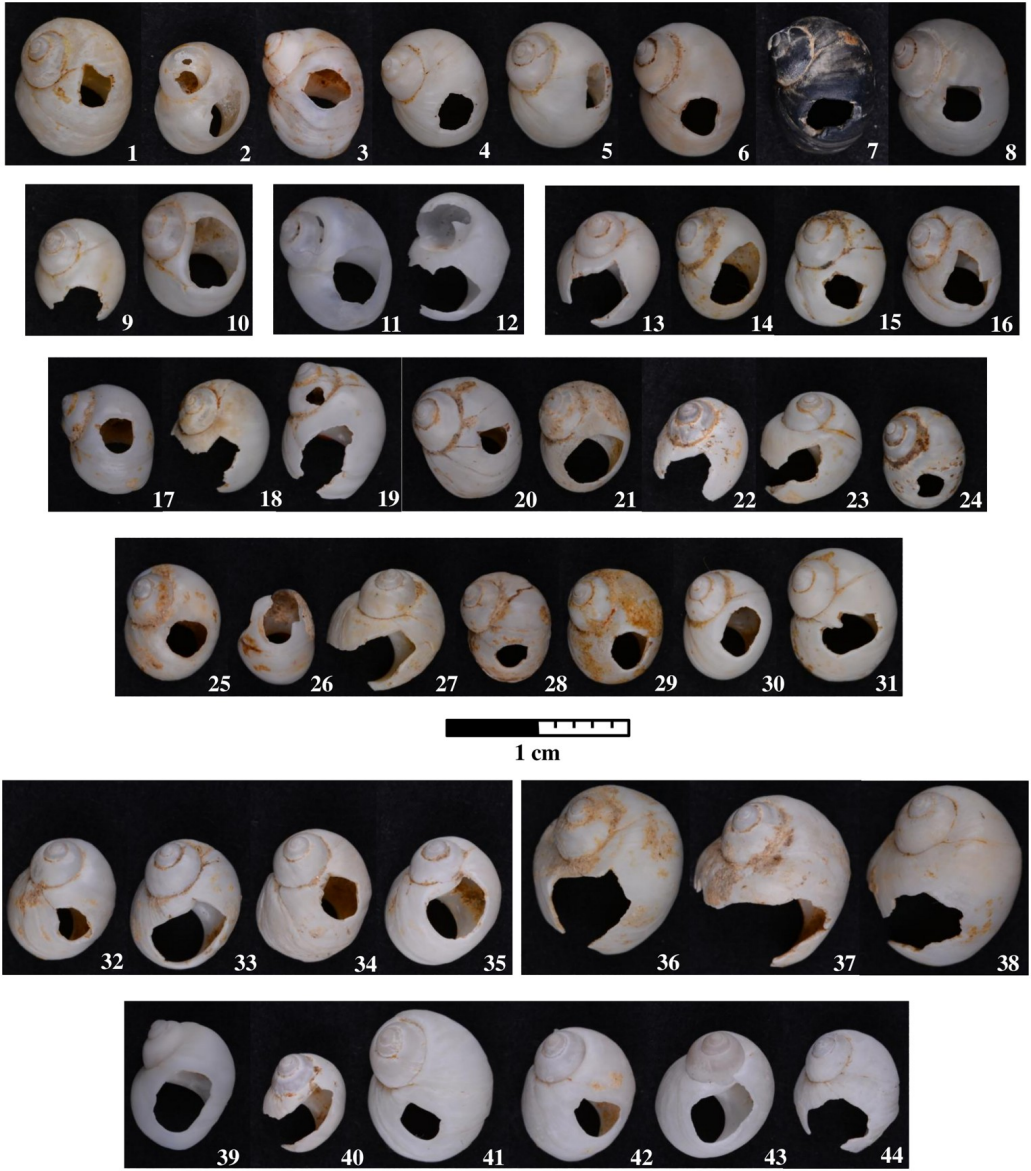
*Lithoglyphus naticoides* and *apertus* perforated shells discovered at Poiana Cireșului. *Lithoglyphus naticoides* (1–35; 39–44) and *Lithoglyphus apertus* (36–38) found in 2004 (1–10), 2006 (11–12), 2016 (13–31; 36–38), 2017 (32–35) and 2018 (39–44).

**Fig 3:**
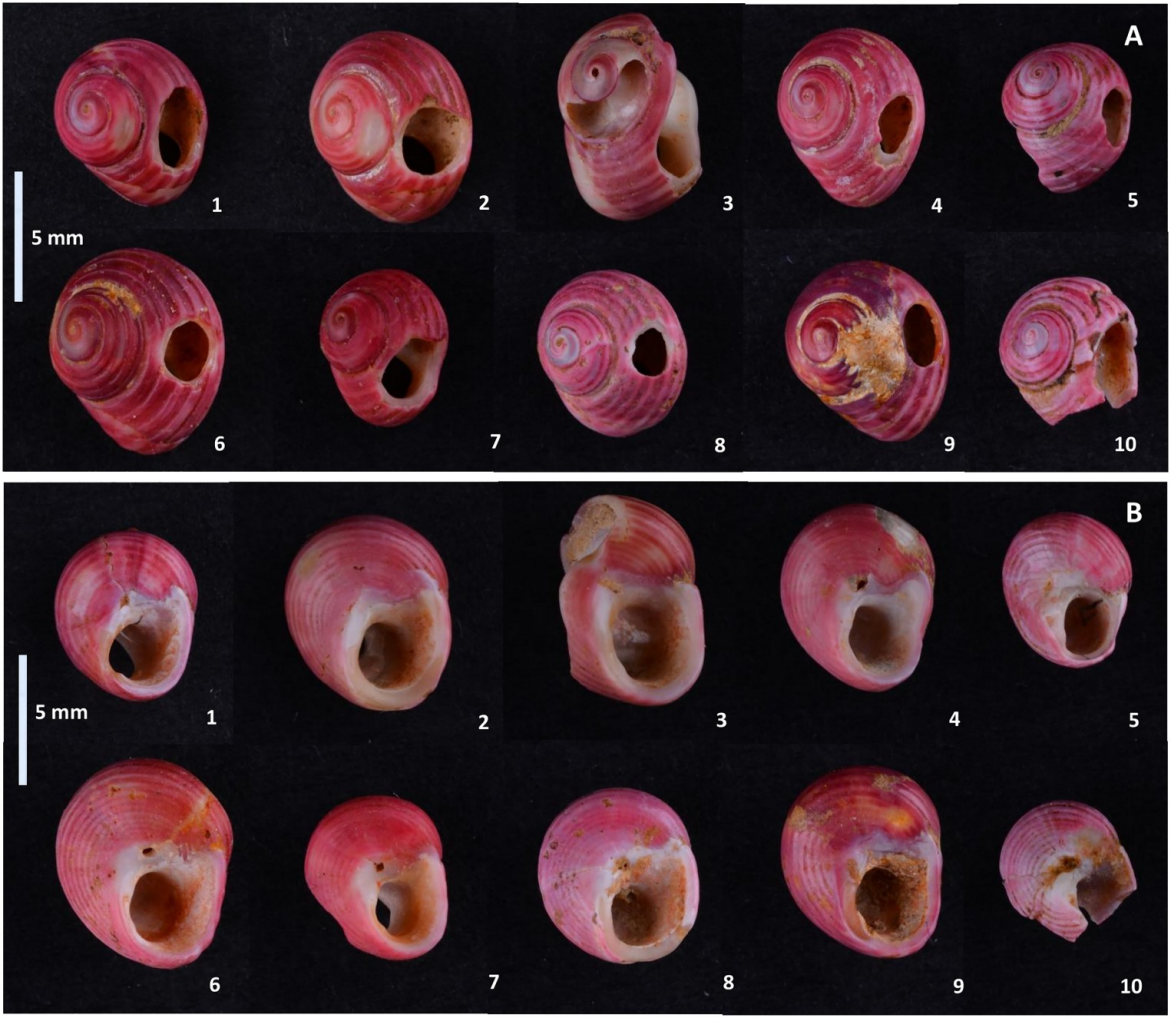
*Homalopoma sanguineum* perforated shells discovered at Poiana Cireșului. (A) Images of the dorsal surface. (B) Aperture of shell.

The first perforated shells were discovered at Poiana Cireșului in 2004 (10 specimens) and 2006 (two specimens) in section V. These were attributed to *Lithoglyphus naticoides* (Fig 2). The 10 shells found in 2004 were arranged over tens of cm^2^; therefore it was assumed that they were part of a single necklace [21]. The continuation of excavations in 2018, in the same section, led to the discovery of six specimens of *L*. *naticoides* concentrated in only one square meter. During the 2016 archaeological campaign, 10 perforated shells of *Homalopoma sanguineum* were recovered in section XII, a Mediterranean species. These shells could only have come from very remote distances (Fig 3). In the same section, another 22 perforated shells of *Lithoglyphus* (19 *naticoides* and 3 *apertus*) were recovered. Four more perforated shells of *L*. *naticoides* were found in 2017. No unperforated specimens of the two species were found at Poiana Cireșului although one may assume that the *Lithoglyphus* were of local origin.

**Fig 4:**
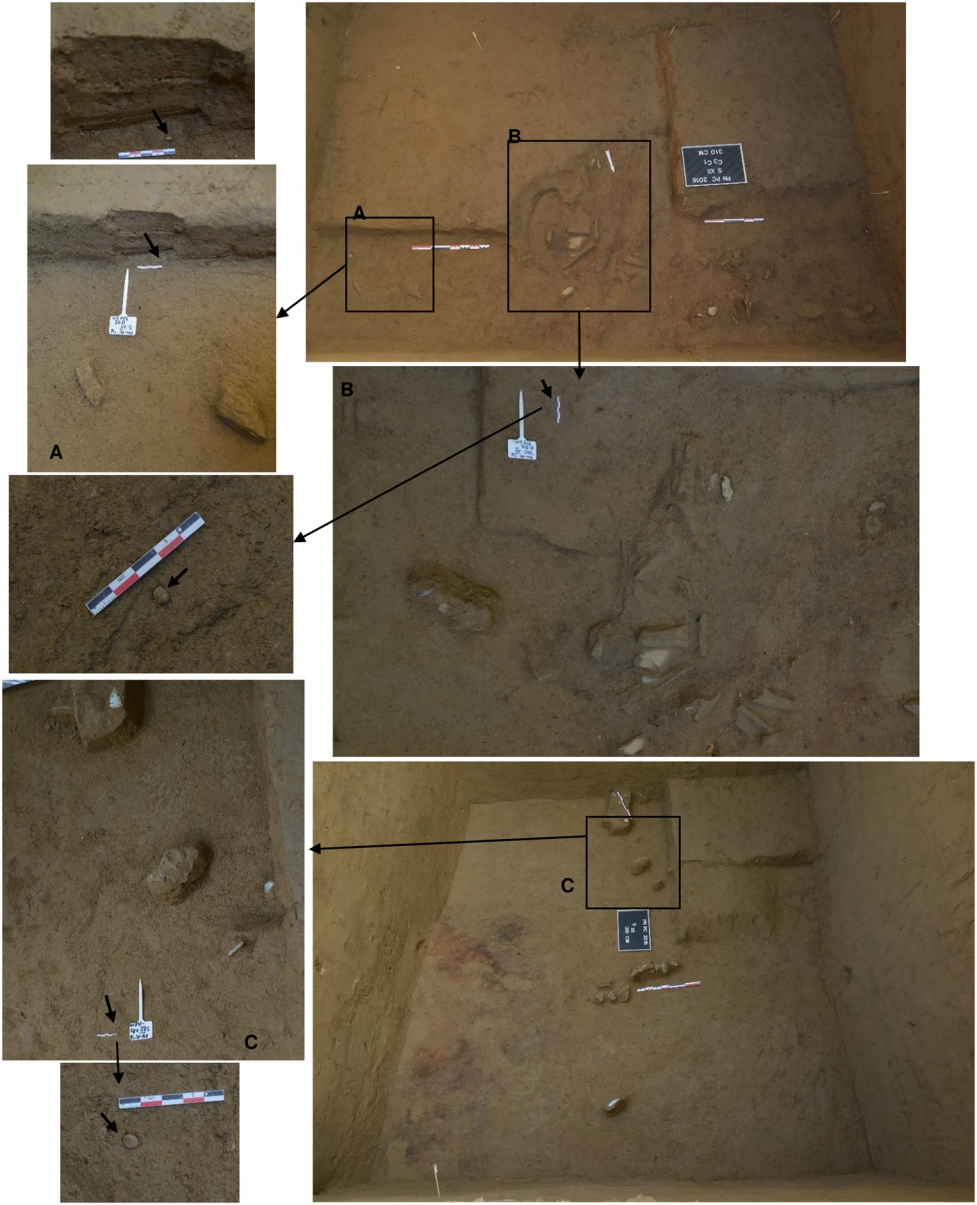
Images of the in situ discovery of the perforated shells. (A), (B) *Lithoglyphus naticoides* perforated shells. (C) *Homalopoma sanguineum* perforated shell.

**Fig 5:**
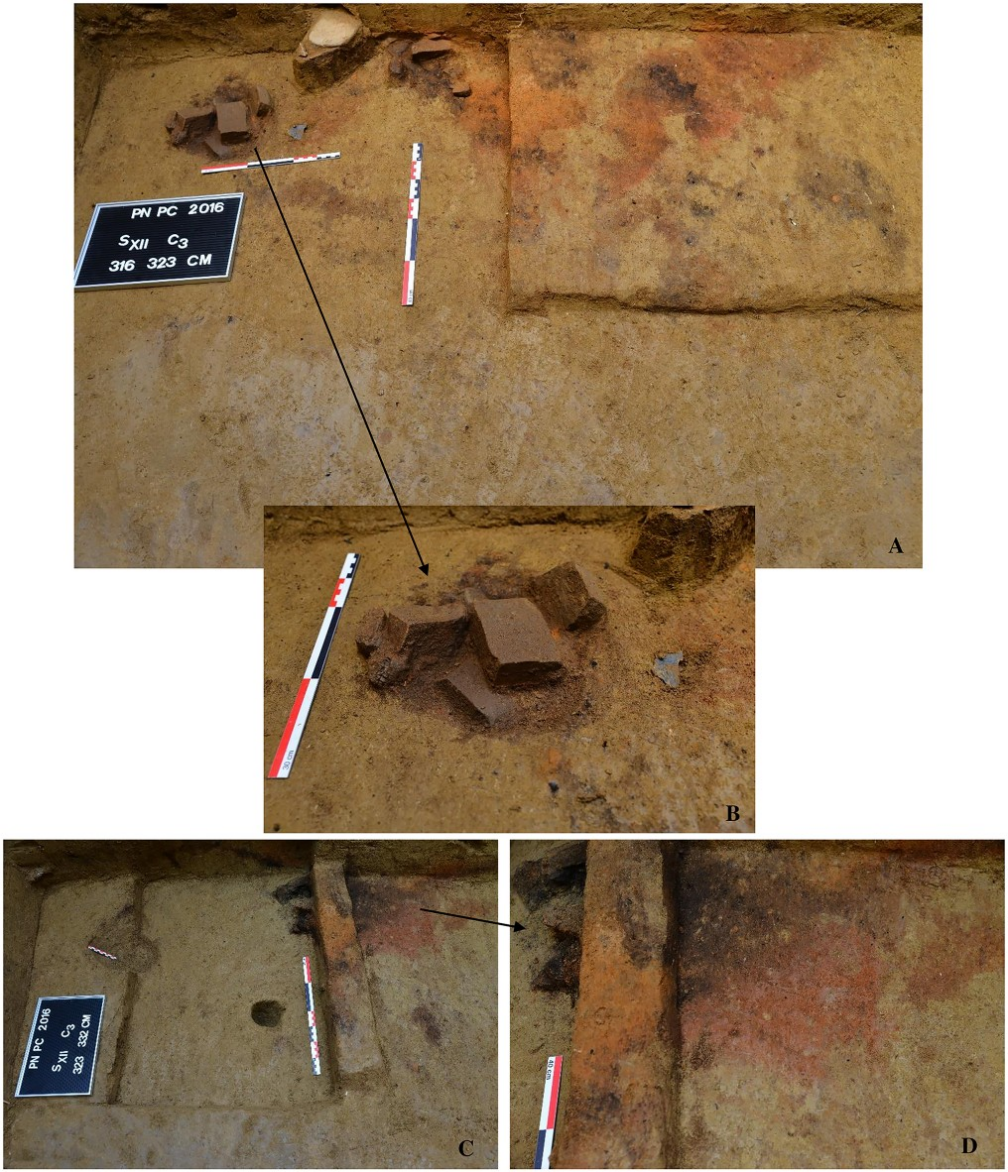
Images during the excavation of the hearth in section XII at Poiana Cireșului. (A) first stage of the excavation. (B) detail of the pit filled with limestone fragments. (C, D) second stage of the excavation.

The perforated shells of *Homalopoma sanguineum* and most of the *Lithoglyphus* were found during the 2016 excavation campaign. They were gathered in a small area of only nine m^2^ (Fig 4). The density of the materials discovered is small in relation to the excavated surface and is represented by a few concentrations of bone fragments of large herbivores (Bos/Bison) and a few lithic materials (Fig 4). On the northern side of the section, a shallow hearth, 20 cm thick, with well-preserved and intense burning traces, was delineated (Fig 5). Near the hearth, there is a pit with a maximum diameter of 30 cm that had been filled with angular limestone fragments with traces of burning (perhaps a water-heating pit) (Fig 5B). A few other small pits may be part of a dwelling structure (Fig 5C).

The spatial distribution of the perforated shells points to a concentration of *Homalopoma sanguineum* specimens in only three adjacent squares of one m^2^ each in section XII, while the *Lithoglyphus* is evenly distributed—particularly around the two hearths found in sections V and XII (S1 Fig).

Although the nature of the sediment and its humidity were an impediment, half of the perforated shells were found in the excavation; the others were found during sieving. Both excavation method and the sieving process (mesh of 3 mm at most) were crucial to the recovery of perforated shells. This may also explain the complete lack of ornaments in the other sites of the Bistrița valley, which have to be investigated in the future by applying the same methods of excavation and recovery as at Poiana Cireșului.

The items are very well preserved except for a few traces of carbonates on the surface of some shells (Figs 2 and 3).

## Methods

Repository information: the specimens are housed at the “Princely Court” National Museum Târgovişte, section Museum of Human Evolution and Technology in Paleolithic. The identification numbers of the specimens analyzed are LXIX 49251–49278 for the *Lithoglyphus* collection and LXIX 49279–49286 for the *Homalopoma sanguineum* collection. No permits were required for the artefacts’ study as first author (E.-C. N.) is responsible for the excavation and the scientific activity at the site of Poiana Cireșului; all authors are members of the research team. For the archaeological excavations, the permits were obtained from the Ministry of Culture and National Identity, Romania (National Archaeological Repository code 120735.04).

During the analysis, each shell of the two species was conventionally assigned a number found in the tables and figures of this paper. The perforation method and use-wear analysis for 12 of the *Lithoglyphus naticoides* specimens as well as the experiments to determine their identify have been published [21]. Observations on the specimens found in recent years have been compared to previous results [22, 23, 24]. Several sources were used to identify the perforation methods employed on the *Homalopoma sanguineum* shells. These describe the main techniques such as direct and indirect percussion, pressure, scratching, grinding, and rotation [25, 26, 27, 28, 29]. During the analysis, we sometimes considered perforations to be made by combining several methods. Use-wear marks were particularly determined via microscopic analyses—visible traces were remarked on the perforation rim (deformations, rounding, polish), on the shell surface (micro-striations, polish, faceting, smoothing), and on the aperture (deformations, polish, smoothing); these were then compared to published sources—mainly with those on marine gastropod shells [30, 31, 32, 33, 34, 35, 36]. Also, observations were made in terms of taphonomic alterations for each shell such as the presence or absence of the original pigmentation, littoral action, recrystallization, erosion, fossilization, burning, etc. [34, 37].

All shells were studied with microscopy. An STM 8-T stereomicroscope equipped with a Nikon camera and a Keyence VHX-600 (x 200 magnification) optical digital microscope was used. The photographs were captured with a Nikon D-600 camera equipped with the following lenses for macro photography: Tamrom SP 90 mm, f 2,8, Di VC USD Macro 1:1, Makro Planar Zeiss Milvus 2/100 and Sigma 105 mm DG Macro HSM lenses.

## Results

The shells found at Poiana Cireșului have the following malacological features:

*Lithoglyphus naticoides* is a gastropod with an oval shell and an irregularly striated fine surface. It is grayish white with a matt glossy aspect. The maximum size is 8 mm high and 7 mm wide. The shell has five relatively convex anfracts that grow abruptly; the upper part ends with a flattened ridge. The biotope of this snail is mainly in rivers and less in freshwater lakes; it fixes itself to stones or other remains at the bottom of the water. It is found in the Pontic Basin, in the Dniester, Dnieper, Don, and Donets rivers and in some tributaries in the Danube Basin [38].
*Lithoglyphus apertus* is slightly larger: 13 mm high and 14 mm wide. The shell is globular, grayish-white, and finely striated. The spire is widely conical—very low and flattened—with a prominent apex. Of the five anfracts, the last is very big. It lives on the rocky bottom of some rivers and large streams. Today it can occur in the Upper Danube, in the Sava river, etc. [38].
*Homalopoma sanguineum* is a red gastropod that belongs to the *Turbinidae* family (subclass Prosbranchia, Archaeogastropoda order). It currently lives in the Mediterranean Sea; this species likely also existed during the cold Würmian periods. The cold climate at this stage did not cause the species to disappear [27].

The perforated *Lithoglyphus naticoides* shells of Poiana Cireșului come mostly from mature specimens, but some smaller (younger) specimens were also used (Table 2). Taphonomically, the only traces noticed on the shell surface are deposits of calcium carbonates—particularly on shells from section XII (Fig 2). One of the shells (no. 7) is black because it was burnt. It is difficult to say whether this burning was accidental or deliberate considering that it was found near the hearth in section V (S1 Fig). The maximum height of most specimens exceeds 6.3 mm, while the maximum basal diameter is over 5 mm. The largest specimens have a height of 7.7 mm, and the maximum diameter is 5.8 mm.

**Table 2:** Dimensions of the *Lithoglyphus naticoides* (1–35, 39–40) and *L*. *apertus* (36–38) shell beads.

The conventional number of each shells, context, coordinates etc.	Height (maximum diameter) in mm	Width (maximum basal diameter) in mm
**1** Poiana Cireșului, 2004, S-V, square A1-A2, 375 cm	7.5	6.9
**2** Poiana Cireșului, 2004, S-V, square A1-A2, 375 cm	- (broken apex)	6.1
**3** Poiana Cireșului, 2004, S-V, square A1-A2, 375 cm	7.6	6.7
**4** Poiana Cireșului, 2004, S-V, square A1-A2, 375 cm	7.2	6.4
**5** Poiana Cireșului, 2004, S-V, square A1-A2, 375 cm	7.7	6.6
**6** Poiana Cireșului, 2004, S-V, square A1-A2, 375 cm	7.8	6.9
**7** Poiana Cireșului, 2004, S-V, square A1-A2, 375 cm	7.7	7.0
**8** Poiana Cireșului, 2004, S-V, square A1-A2, 375 cm	7.8	6.7
**9** Poiana Cireșului, 2004, S-V, square A1-A2, 375 cm	- (broken perforation)	-
**10** Poiana Cireșului, 2004, S-V, square A1-A2, 375 cm	6.9	5.9
**11** Poiana Cireșului, 2006, S-V, square E-2, 410 cm	7.5	6.9
**12** Poiana Cireșului, 2006, S-V, square E-2, 410 cm	- (broken perforation and apex)	-
**13** Poiana Cireșului, 2016, S-XII, square A-2, 345 cm	- (broken perforation)	5.1
**14** Poiana Cireșului, 2016, S-XII, square B-2, 360 cm	6.7	4.8
**15** Poiana Cireșului,2016, S-XII, square C-1, 360 ± 5 cm	6.9	5.1
**16** Poiana Cireșului, 2016, S-XII, square B-2, 365 cm	6.6	5.2
**17** Poiana Cireșului, 2016, S-XII, square A-3, 360 cm	6.3	4.8
**18** Poiana Cireșului, 2016, S-XII, square C-1, 365 cm	6.4	- (broken perforation and aperture)
**19** Poiana Cireșului, 2016, S-XII, square B-2, 361 cm	7.1 (broken perforation)	5.1
**20** Poiana Cireșului, 2016, S-XII, caroul B-1, 365 cm	7.2	5.9
**21** Poiana Cireșului, 2016, S-XII, square B-3, 357 cm	6.7	5.2
**22** Poiana Cireșului, 2016, S-XII, square A-2, 355 cm	- (broken perforation)	4.8?
**23** Poiana Cireșului, 2016, S-XII, square C-1, 360 ± 5 cm	6.4? (broken perforation)	5.1
**24** Poiana Cireșului, 2016, S-XII, square B-3, 355 cm	5.9	4.5
**25** Poiana Cireșului, 2016, S-XII, square, C-3, 360 ± 2 cm	6.2	5.0
**26** Poiana Cireșului, 2016, S-XII, square, C-3, 360±2 cm	- (broken perforation)	-
**27** Poiana Cireșului, 2016, S-XII, square A-2, 358 cm	- (broken perforation)	4.8?
**28** Poiana Cireșului, 2016, S-XII, square B-3, 369 cm	6.8	5.0
**29** Poiana Cireșului, 2016, S-XII, square C-3,369–368 cm	7.7	5.5
**30** Poiana Cireșului, 2016, S-XII, square C-3, 355 ± 5 cm	6.5	4.8
**31** Poiana Cireșului, 2016, S-XII, square B-3, 358 ± 3 cm	7.3	5.8
**32** Poiana Cireșului,2017,S-XIV, square B-3, 365–373 cm	7.2	5.0
**33** Poiana Cireșului,2017, S-XIV, square B-3, 365–373 cm	7.5	5.4
**34** Poiana Cireșului, 2017,S-XIV, square B-3, 365–373 cm	7.5	5.8
**35** Poiana Cireșului, 2017,S-XIV, square B-3, 365–373 cm	7.1	5.5
**36** Poiana Cireșului, 2016, S-XII, square C-1, 350 cm	- (broken perforation)	8.5
**37** Poiana Cireșului, 2016, S-XII, square A-2, 345 cm	10.1 (broken perforation)	7.9
**38** Poiana Cireșului, 2016, S-XII, square B-3, 357 cm	9.1 (broken perforation)	-
**39** Poiana Cireșului, 2018 S-V, square A-4, 380 cm	7.0	5.1
**40** Poiana Cireșului, 2018 S-V, square A-4, 380 cm	- (broken perforation)	4.5
**41** Poiana Cireșului, 2018 S-V, square A-4, 381 cm	8.2	6.5
**42** Poiana Cireșului, 2018 S-V, square A-5, 381 cm	7.4	5.3
**43** Poiana Cireșului, 2018 S-V, square A-4, 382 cm	7.9	5.4
**44** Poiana Cireșului, 2018 S-V, square A-4, 382 cm	- (broken perforation)	5.3

Despite their apparent fragility, only 15 of the 44 specimens are fragmented: Of the 13 shells, only the perforation is broken, one have a broken apex, and another one has a fractured perforation and apex (S2B Fig). Some of the shells with broken perforation still have traces of rounding and polishing on the perforation rim (S2 Fig). The position of fractures towards the outer lip as well as the preservation of use-wear marks point to the fracturing of perforation during wearing [39]. Five specimens have accidental holes on the surface—most of them (4) are located on the ventral face and the body whorl; in only one case is it on the dorsal side, located on the spire, i.e., the surface between the perforation and apex (S2B Fig). Three shells have traces of faceting around the holes. At least a part of the accidental holes resulted from shell friction and hitting against one another during wear as shown by the experiments made on *L*. *naticoides* shells [22].

**Fig 6:**
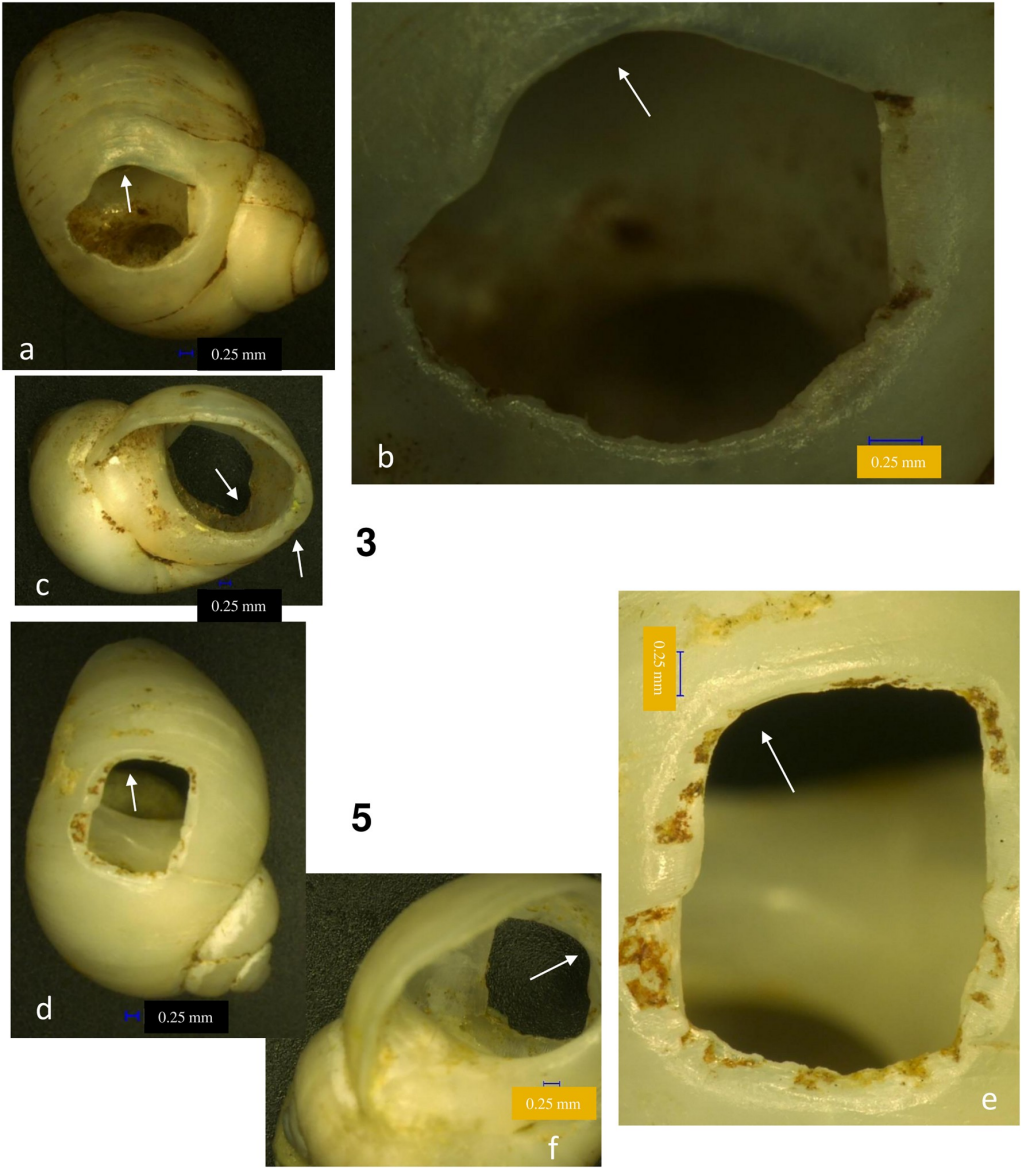
*Lithoglyphus naticoides*–use-wear traces for shells 3 and 5. (a, d) General outline and perforation deformation. (b, e) Detail of perforation (the arrow marks the traces of smoothing and polishing). (c) Perforation and aperture deformation. (f) Perforation deformation.

**Fig 7:**
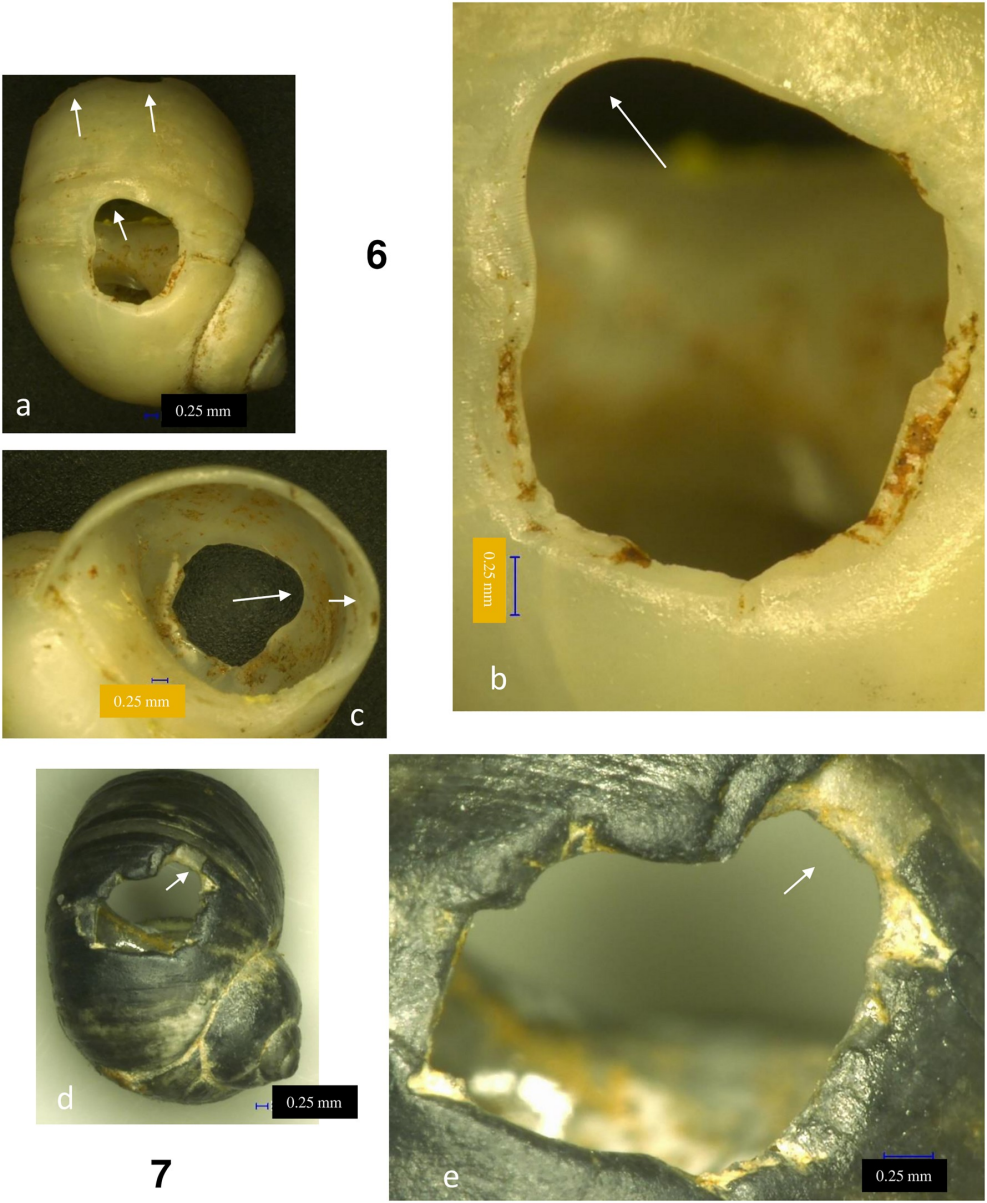
*Lithoglyphus naticoides*–use-wear traces for shells 6 and 7. (a) General outline and perforation deformation. (b, d, e) Detail of perforation (the arrow marks the traces of smoothing and polishing). (c) Perforation and aperture deformation.

All *Lithoglyphus* shells were perforated by pressure from the inside of the shell: The perforation has a sub-rectangular or irregular contour and micro-chipping on the outer part [21, 26, 34] (Figs 6d, 6e and 7d; S2A Fig). The perforation location is uniform throughout the entire assemblage and is constantly positioned on the last whorl (dorsal side of shells) (Fig 2). This area corresponds very well to the aperture. Most perforations have deformations, rounding and polish in the hanging area (Figs 6 and 7). Marks on perforations correspond to small aperture deformations (Figs 6c and 7a and 7c) and, alongside the traces on the shell surface (smoothed and polished area, holes), prove they were strung and worn, bead-to-bead, with the apex down.

The three *Lithoglyphus apertus* specimens have the perforation broken in the same manner noted on the *L*. *naticoides* specimens. This is most likely fracturing during wear.

The *Homalopoma sanguineum* perforated shells generally come from mature specimens. Most have a maximum diameter of over 6.40 mm and a minimum of at least 5.20 mm. The largest of the specimens is 8.14 mm (maximum diameter) by 6.99 mm (minimum diameter), while the smallest has a maximum diameter of 5.78 mm and a minimum of 4.96 mm (Table 3). Of the 10 specimens found, only two shells are fragmented (Fig 3). One was damaged both by littoral action [34, 37] and during manufacture (Fig 3A3). It was worn fragmented as shown by the rounding and polish marks on the hole. The other fragmented shell is also the smallest specimen (Fig 3A10). It has a broken perforation with the fracture morphology that is similar to those noted on the *Lithoglyphus* specimens. All shells preserve the original color. The only post-depositional traces noted are small areas with calcium carbonates on the surface. This is obvious in only one specimen (shell 9).

**Table 3:** Dimensions of the *Homalopoma sanguineum* shell beads.

Number and Coordinates	Maximum height in mm	Maximum diameter in mm	Maximum diameter of the perforation in mm	Minimum diameter of the perforation in mm
1: Square B-2, depth 365 cm	6.70	5.93	3.24	1.49
2: Square B-2, depth 365 cm (X-75, Y-46)	7.42	6.22	2.65	2.21
3: Square A-2, depth 355 cm (X-52, Y-20) Fragmented	-	-	-	-
4: Square A-2, depth 355 cm (X-52, Y-20)	7.18	5.96	2.36	1.38
5: Square A-1, depth 360 cm (X-13, Y-28)	5.78	4.96	-	-
6: Square A-2, depth 355 cm	8.14	6.99	2.40	1.62
7: Square A-2, depth 355 cm	6.41	5.23	2.82	1.74
8: Square A-2, depth 345 cm	6.45	5.67	1.87	1.63
9: Square A-2, depth 355 cm (X-55, Y-30)	7.34	6.34	2.51	1.19
10: Square A-1, depth 359 cm Fragmented	-	-	-	-

There is great consistency in terms of the perforation position in the *Homalopoma sanguineum* shells. In all the specimens, the hole was made in the middle of the umbilicus, i.e. on the last anfract or whorl. It is located near the suture between the umbilicus and the whorl preceding it. This area is called E2 by Taborin [27, 30]. The hole was constantly positioned in the same area of the shell and communicated directly with the aperture to facilitate the insertion of the suspension thread. Perforation sizes are quite similar (Table 3): Large diameters vary between 3.24 mm and 1.87 mm, while minimum diameters are between 2.21 mm and 1.19 mm.

**Fig 8:**
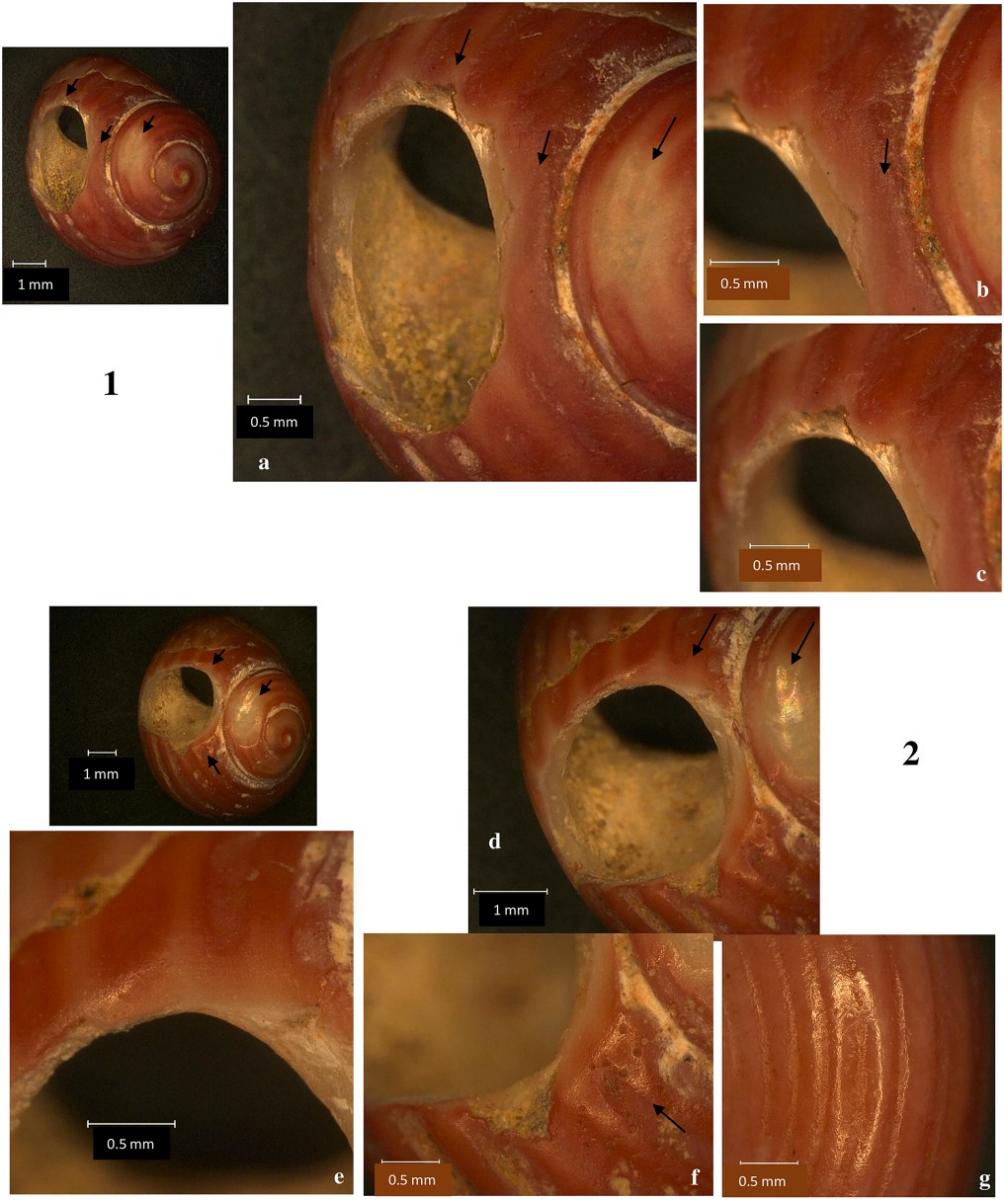
*Homalopoma sanguineum*–perforation method and use-wear traces for the shells 1 and 2. (a, d) Detail of perforation (the arrow marks the traces of smoothing and polishing on perforation rim, grinding traces, and area around the apex smoothed by wearing). (b, f) Grinding traces. (c, e) Use-wear on perforation. (g) Polish and striation on the dorsal side of the shell.

**Fig 9:**
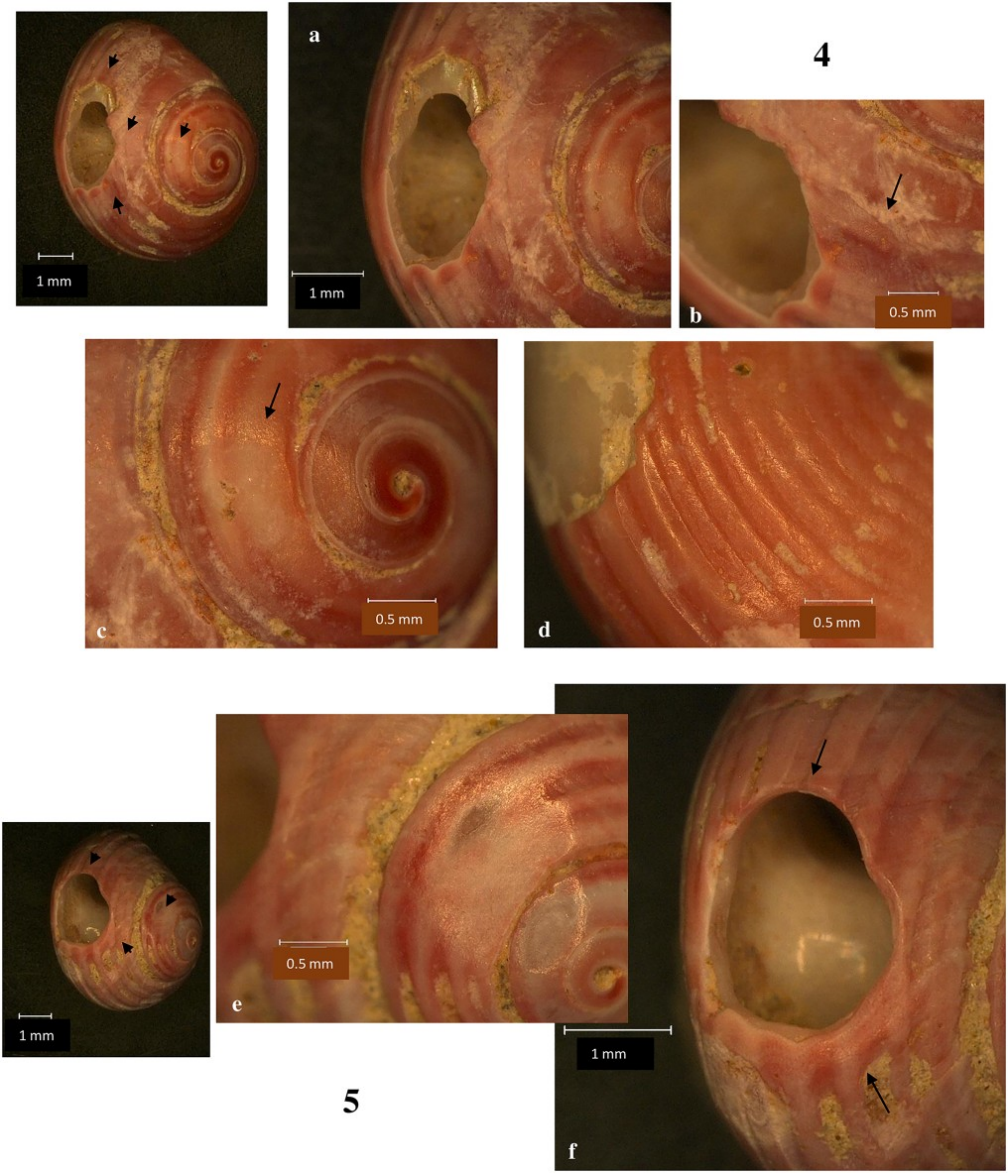
*Homalopoma sanguineum*–perforation method and use-wear traces for the shells 4 and 5. (a) Polishing on perforation. (b) Grinding traces. (c, e) Area around the apex smoothed by wearing. (d) Polish and striation on the dorsal side of the shell. (f) Use-wear on perforation and grinding traces.

**Fig 10:**
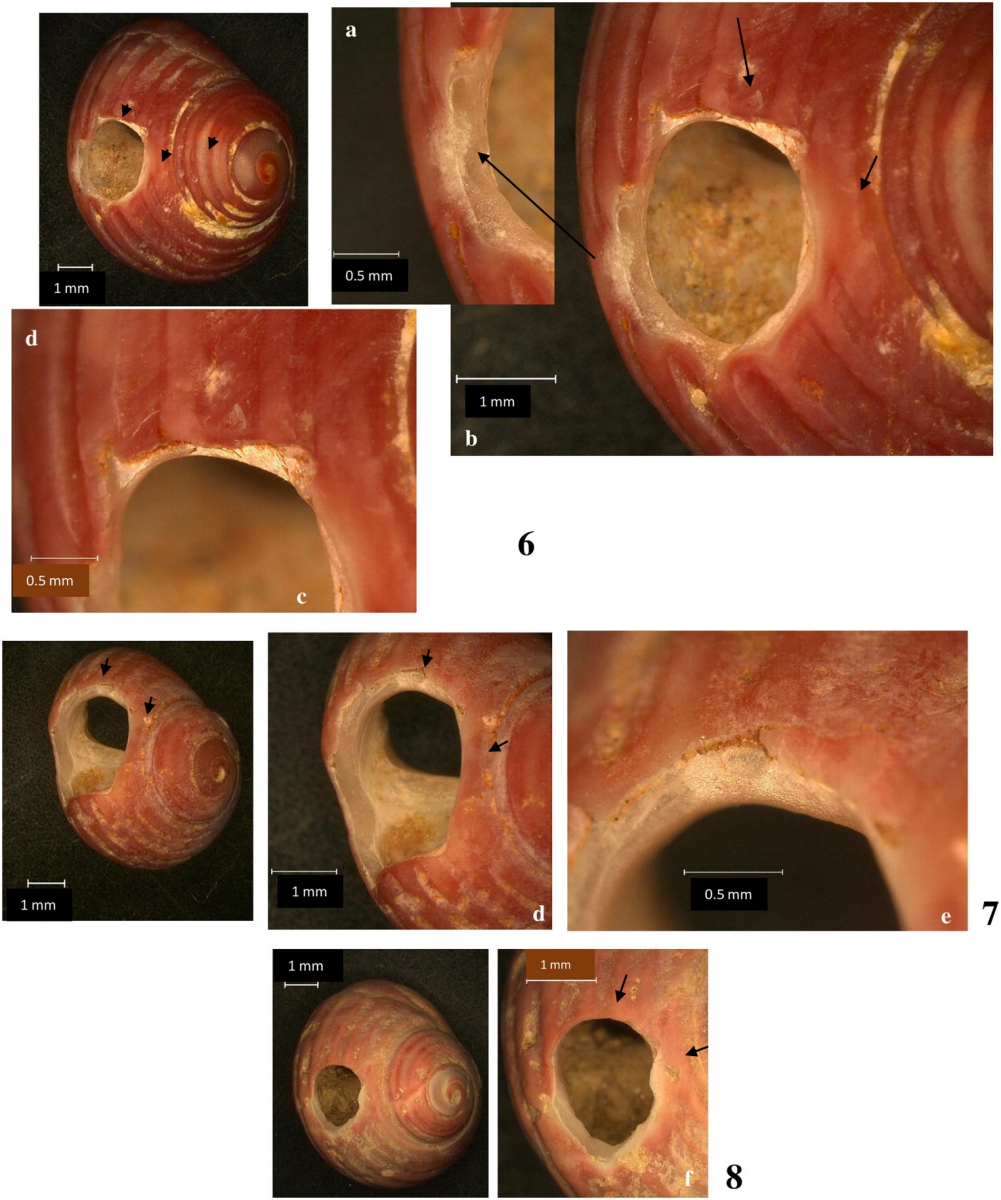
*Homalopoma sanguineum*–perforation method and use-wear traces for the shells 6, 7 and 8. (a) Scratching marks. (b, d, f) Use-wear on perforation and grinding traces. (c, e) High polish on perforation.

According to microscopic observations, the shells were perforated with grinding and gouging (motion that involve pressure and rotation) [25, 29]. Most of the specimens have preserved the traces produced by grinding around the hole. The holes are characterized by flattening of surfaces and striation (Figs 8b, 8f and 9b, 9f and 10b, 10d). Gouge marks are noted on all shells. They have external wedging (S2C Fig), scratching (Fig 10a), and blunt edge perforations (Figs 8–10) [29]. The morphology of the holes is circular or slightly oval with regular contours (S 2C Fig). In fact, grinding was used for shell thinning while pressure and rotation were employed to complete the perforation. The combined use of grinding and gouging was described for very thick-walled shells [25, 28] as are the *Homalopoma sanguineum* shells. *Homalopoma sanguineum* shells were worn by suspension—there is a clear rounding in the area that came in permanent contact with the suspension thread and intense polishing (Figs 8–10). The traces noted on the perforation rim correspond to small deformations noted on the aperture. Furthermore, areas with striations and polish have been noted on the dorsal side of shells (Figs 8g and 9d). There are very smooth and polished parts between the perforation and the apex where the periostracum and a part of the ostracum are removed (Figs 8a, 8d and 9c, 9e). Therefore, shells have utilization marks on both the dorsal and ventral sides. This most likely resulted from the beads’ friction against each other. The suspension manner resembles that noted on the *Lithoglyphus* shells where they are strung in the shape of necklaces or other similar ornaments. All traces point to prolonged wearing of shells.

## Discussion

Unlike some species of marine gastropods, like *Tritia* sp. (common species of the Black Sea and of the Mediterranean), *Homalopoma sanguineum* has never been mentioned in the Black Sea. It is not found in the catalogue of gastropods of Romania that includes the studies from the western and northern coasts of the Black Sea [38, 40]. Recent updating of the database of marine mollusks on the Turkish coast, which includes the Black Sea, the Aegean Sea, the Sea of Marmara, the Levantine Sea, the Bosphorus and Dardanelles straits, is indicative of the distribution of mollusks in this area [41]. Not only has *Homalopoma sanguineum* not been encountered in the Black Sea, but it has always been absent from the Bosphorus strait that represents the connection with the Sea of Marmara where this species currently exists. In fact, of all the above-mentioned seas, the Black Sea has the fewest species of mollusks [41].

**Fig 11:**
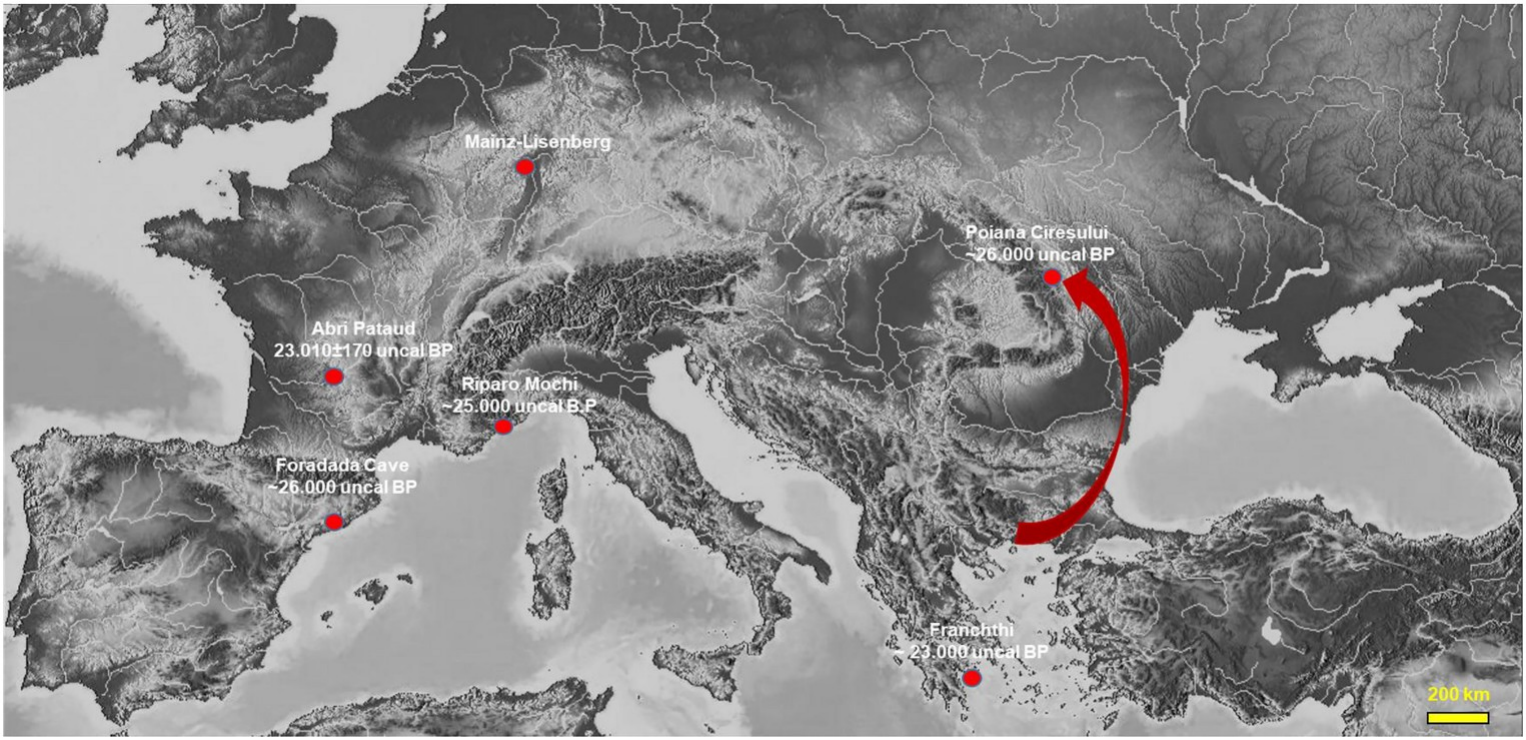
Gravettian settlements with discoveries of perforated *Homalopoma sanguineum* and a possible route of movement from the Aegean Sea to the east of the Carpathians. (modified after https://maps-for-free.com/).

Considering that *Homalopoma sanguineum* is a Mediterranean species, the Sea of Marmara and the Aegean Sea are the closest and most plausible sources in terms of the distance to the Poiana Cireșului site. The route of populations can be easily established along the Black Sea coast to then follow the watercourses of the Prut or the Siret and further on along the Bistrița valley (Fig 11).

Some features of the Black Sea coast need more explanation. The beginning of the Würmian glacial stages led to an abrupt decline in the Black Sea (100–110 m). In fact, during this period the Black Sea again became a lake with semi-salty water and Caspian fauna; connections with the Mediterranean were cut [42, 43]. The Black Sea’s water level rose again between 40,000 and 25,000 BP during the Surozhian interstadial as shown by the increase in water salinity and the emergence of Mediterranean marine fauna. These prove the connection of the two seas through the Bosphorus strait. There were relatively minor changes during the two Surozhian sub-phases. The Black Sea’s level was about 10 m lower than nowadays during the first phase. During the second phase it reached the level of today and, in some areas, it slightly exceeded it. On the southeastern coast of Romania, cliffs eroded by waves were found approximately 12–38 m deep, which points to a slightly lower level of the shore compared to the present day [42, 43]. The sandy deposits of ca. 28,500–25,000 years BP ago indicate a position of the western shore of the Black Sea relatively close to the current Danube delta [44]. Instead, northwest of the Black Sea, the palaeo-Prut, the palaeo-Dnieper and the palaeo-Bug formed a deltaic relief that descended to the south from 180 to 80 m deep when compared to the current sea level [45]. During the existence of the Neoeuxinian lake, a terrace dug by waves could be noticed 100 kilometres along the Romanian shore at 98–115 m deep [43].

No discoveries of Paleolithic settlements with *Homalopoma sanguineum* have yet been made in relation to the route along the Black Sea as far as the Aegean Sea. There are very few Paleolithic sites in Greece in which *Homalopoma sanguineum* specimens have been found (Fig 11). At Franchthi, in the four levels attributed to the Early Upper Paleolithic, only 14 specimens have been found: Four are perforated, and six lie in Gravettian levels (layers R and S1) [46]. As three of the perforated shells come from Aurignacian levels [47], this means that only one perforated specimen is in one of the two Gravettian layers. A recent dating of the Gravettian level (layer R) has indicated an age of 23,510 ± 90 BP [47]. At Klissoura Cave 1, no *Homalopoma sanguineum* specimen has been found in Gravettian levels although this species is common in Aurignacian occupations [48]. Also, at Klithi and Boila, *Homalopoma* shells have only been found in Late Epigravettian occupations [49]. In settlements east of the Mediterranean including Turkey, Paleolithic levels have revealed no specimens of *Homalopoma sanguineum* [34, 50].

Elsewhere in Europe, Gravettian settlements where perforated shells of *Homalopoma sanguineum* have been mentioned are quite rare (Fig 11). Most of the shells were discovered in sites that are closer to the Mediterranean coasts. At Riparo Mochi in Italy, approximately 200 specimens were recovered in levels D and F [51]. Only level D is attributed to the Gravettian, and new dating suggests an age around 25,000 BP while level F is dated to between 26 and 27 ka BP and is attributed to the Aurignacian [52]. Also, five unperforated shells were discovered at Grotta della Serratura in the level 11 dated to 24.380 ± 1530 (Beta– 88907) [53]. More than 100 perforated specimens of *Homalopoma sanguineum* have been recently discovered in the Foradada Cave (Spain) in a level dated around 30–31 ka cal. BP attributed to the Early Gravettian [54]. In inland sites, the number of *Homalopoma* shells found is very small. At Abri Pataud (France), in the Perigordian VI (N3), only one perforated shell of the species *Homalopoma sanguineum* has been found [27]; the settlement is located 200 km from the Mediterranean shore [55], and that particular level is dated to 23,010 ± 170 BP. At Mainz-Lisenberg on the Rhine River, in an occupation attributed to the Perigordian VI/VII, 15 specimens of *Homalopoma sanguineum* perforated shells were found [56]. We should consider that this site and the Mediterranean coast are 800 km apart, which made E. Alvarez Fernández [55] speak about the existence of a complex network of contacts between human groups; however, he dismissed the idea of direct displacements of these groups across such an appreciable distance. Except for the Mainz-Lisenberg settlement, no other Gravettian sites with *Homalopoma sanguineum* have been found in Central Europe.

The use of *Lithoglyphus* shells, found in great number at Poiana Cireșului, is also interesting. There are no specimens of perforated shells attributed to this species in other Gravettian settlements in this area although the species is widespread in the Danube basin and the Pontic-Caspian area. One may refer to the limited occurrence of the species, although it has been attested in deposits that are older and even contemporaneous with those of Poiana Cireșului. Such an example was mentioned in the Danube Basin of Germany in the deposit of Sesselfelsgrotte II, layer N dated to 56,653 ± 6,934 cal. B.P. and in the upper layer O [57]. At Ripiceni Izvor (north-east of Romania) shells were identified in the sands covering the gravels of the 13-m relative elevation terrace of the Prut, over which was located the Palaeolithic levels, dated to over 46,400 + 4,700/ - 2,900 B.P. (GrN 11.230), [58]. Furthermore, unperforated shells of *Lithoglyphus naticoides* have been recovered from Gravettian settlements in Central Europe such as the site of Milovice IV [59] and in Eastern Europe at Buran Kaya 3 [60]. The only mention of perforated shells of *Lithoglyphus naticoides* in the Early Upper Paleolithic of Eastern Europe is at Siuren I [61], but the specimens were found in an Aurignacian context. In contrast, this species was frequently used during the Late Epigravettian as is the case of Yudinovo, Russia [62], Riparo Biarzo, Italy [63], and settlements on the coast of the Adriatic Sea [64, 65] particularly in Epipaleolithic and Mesolithic sites such as those of the Iron Gates Gorges of the Danube [66].

The association of mollusk genera used at Poiana Cireșului (*Litoglyphus* and *Homalopoma*) is not to be found in any other Paleolithic site. This collection of perforated shells does not fit into the general Gravettian traditions in Central and Eastern Europe where other species are used.

In Central Europe, most shells come from fossil deposits and most of them are not perforated; in Eastern Europe, both fossil and marine species from the Black Sea and freshwater species were used.

Central European settlements with Gravettian occupations chronologically close to Poiana Cireșului are suggestive of the uniformity of shells used in the Gravettian in this area. At Dolni Věstonice I, II and III, all the shells found come from tertiary deposits [67]. Around 600 tertiary mollusk shells were discovered in the grave of Brno II; one of the species is *Dentalium* [67]. At Pavlov I, the Tertiary shells were collected near the site [68]. An impressive number of shells were also recovered at Pavlov VI; these are represented by 204 specimens of tertiary mollusks comprising 159 gastropods, 43 scaphopods, and two bivalves of which only 32 were perforated [68, 69]. Approximately 100 specimens of *Dentalium* were found alongside other mollusks used as adornments (*Melanopsis*, *Terebrarium*) at the Grub/Kranawetberg settlement (Austria) in the main and upper level. These were collected from the old marine sedimentary deposits around the site [70]. Perforated shells of *Glycimeris* were discovered at Ollersdorf/Heidenberg (Austria)—a settlement located near the Grub/Kranawetberg site [70]. This species had also been mentioned at the settlement of Geißenklösterle and at Hohle Fels as well (both in Swabian Jura, Germany) along with ornaments made from snail shells such as *Dentalium* sp. [71].

No specimens of perforated shells from exclusively Mediterranean species were found in the Gravettian settlements of Eastern Europe (Republic of Moldova, Ukraine, Russia) [72]. Certain species such as the *Tritia* sp., allegedly of Mediterranean origin, are actually found in the Black Sea and in the fossil deposits of this region. For example, three specimens of *Tritia* sp. were found in Gravettian I of Poiana Cireșului but they originate from fossil deposits [14]. Also, the *Columbelidae* shell discovered at Kostenki 14 [73] can be derived from the Black Sea [74].

Aside from the species used (*Homalopoma sanguineum*, *Litoglyphus naticoides* and *L*. *apertus*), the shell collection of Poiana Cireșului has other special characteristics as well. All shells have a round shape. Their very small size is particularly surprising: Nearly all sizes are 6 to 8 mm. There is obviously a high degree of selection of shells depending on shape and size. The perforation technique of *Homalopoma sanguineum* shells is also different from the methods described in other settlements. Most assemblages are perforated by direct percussion on the outer side [27, 55] or via indirect percussion from the inside of the shell [46]; the Poiana Cireșului specimens are perforated via both grinding and gouging. Another typical element is the similar manner in which they were worn: Both genera are strung bead-to-bead as indicated by the use-wear marks noted in necklaces, bracelets, or other such ornaments.

In this stage of research, it is very difficult to explain how the perforated *Homalopoma sanguineum* shells ended up at Poiana Cireșului. The fact that all ornaments are finished objects supports two scenarios: either those groups brought their ornaments with them by direct displacement from a Mediterranean area or they were procured within extended networks. For example, based on Mediterranean and Atlantic shells, widespread networks have been established among groups along the Rhine-Rhone axis for Tardiglacial settlements in France, Germany and Switzerland [75].

However, establishing connections among the Gravettian occupations where *Homalopoma* shells have been found is impossible at this moment due to the very small number of sites and chronological differences among them. Except for the Foradada Cave (Spain), which has a similar age as Poiana Cireșului, the Gravettian levels in the other sites are younger. In fact, early Gravettian occupations are generally rare [2]. Italy represents the only region close to the Mediterranean area where sites with early Gravettian occupations are more numerous than in other areas, e.g., Riparo Mochi, Arene Candide, Grotta della Cala, Grotta Paglicci, Grotta Rio Secco, Piovesello [76].

The absence of settlements connecting the Mediterranean Sea to Poiana Cireșului favors the theory of a movement of populations from the Mediterranean area along with their own ornaments. In fact, recent paleogenetic analyses have pointed out that the diffusion of the Gravettian was at least in part due to population movements [77]. The possible Mediterranean origin of these populations seems to be supported by other characteristics of occupation in this site as well. First, the lithic assemblage of Poiana Cireșului displays a very narrow typological range mostly comprising backed tools. This feature has been noticed in some of the early Gravettian occupations in Italy where the collections have been called *Gravettian with backed points* or *Undifferentiated* [78, 79]. Similar lithic assemblages—known as Mediterranean-backed points or Mediterranean-backed blade/bladelets industry—have also been defined in some settlements in Greece such as the Klissoura Cave 1 [80] where a middle-north Mediterranean origin of the Gravettian is presumed. Furthermore, in settlements in Italy and Greece, we note that ornaments were largely made from perforated shells, which is another element that they have in common with Poiana Cireșului. Although the Aegean Sea is the nearest source for shells found at Poiana Cireșului, their provenance from the middle-north area of the Mediterranean cannot be completely ruled out. The older chronology of the Gravettian in some Italian settlements as compared to Poiana Cireșului such as the Paglicci Cave (28,000 BP) [79] or the Rio Secco Cave (29,390–28,995 BP) [81, 82] may represent an argument in this respect. In fact, the existence of a trans-Adriatic corridor linking the Carpathian basin to the Italian peninsula was invoked in the context of the early Gravettian origin [82].

## Conclusions

The symbolic behavior of the Early Gravettian communities of Poiana Cireșului is characterized by the exclusive use of perforated shells from three species: *Homalopoma sanguineum*, *Lithoglyphus naticoides*, and *Lithoglyphus apertus*. The elements that these species have in common are their round shape and the very small sizes of the specimens. As pointed out by Kuhn and Stiner [83], standardization and formal redundancy are essential properties of any system of communication. For Paleolithic adornments, standardization was imposed by the consistent choice of particular natural forms (shells, teeth, claws) and by manufacturing processes. When natural forms are used, people make restrictive selections from a wide range of possibilities. Their selection is obvious for the Poiana Cireșului perforated shells because the uniformity of shapes and sizes is striking. The association of these two genera is unique within the Paleolithic and, alongside the particularities of occupation of Poiana Cireșului, might be proof of social identity.

The presence of a Mediterranean species at the Poiana Cireșului settlement located more than 900 km from the nearest source suggests the connection of communities here with the Mediterranean area as well as the possibility of a movement of populations from the south of the continent to the east of the Carpathians (Fig 11). We believe that a possible route of these populations’ movement may be from the east of the Mediterranean following the Black Sea coast and the great watercourses. The route along the valley of the Siret and then directly by the Bistrița is obviously the straightest. Considering the supply of the Poiana Cireșului communities with sources of lithic raw material from the Prut, we consider this to be a plausible route.

All of these aspects have implications regarding the origin of the Early Gravettian in the east-Carpathian area given that most studies have suggested connections with the Gravettian in Central Europe [1, 2]. Therefore, it is critical to continue studying the areas located along the alleged routes suggested by the places of origin of some species including Mediterranean ones. In this context, the results obtained at Poiana Cireșului prove that, at least in certain regions of Eastern Europe, the origin and diffusion of the Gravettian from the Mediterranean area are an important hypothesis that should be considered.

## Supporting information

S1 Fig Position of the excavated section at Poiana Cireșului. (A) Position of the sections on the topographic map of site. (B) Plan of excavated sections and the position of squares where the perforated shells were found (blue square-*Lithoglyphus naticoides* and *apertus*; red square-*Homalopoma sanguineum* and *L*. *naticoides*; the interrupted line indicates the location of hearths).
(TIF)

S2 Fig Perforation details and use-wear of the shells discovered at Poiana Cireșului. (A) Perforations of the *Lithoglyphus naticoides* shells. (B) Holes on the surface of *Lithoglyphus naticoides* shells. (C) Perforations of the *Homalopoma sanguineum* shells.
(TIF)
