# Supplementary figures and images for: Mobility and social identity in the Mid Upper Paleolithic: New personal ornaments from Poiana Cireșului (Piatra Neamț, Romania)
Source: PLoS One. 2019 Apr 24;14(4):e0214932. doi: 10.1371/journal.pone.0214932 (PMC6481798; doi:10.1371/journal.pone.0214932)

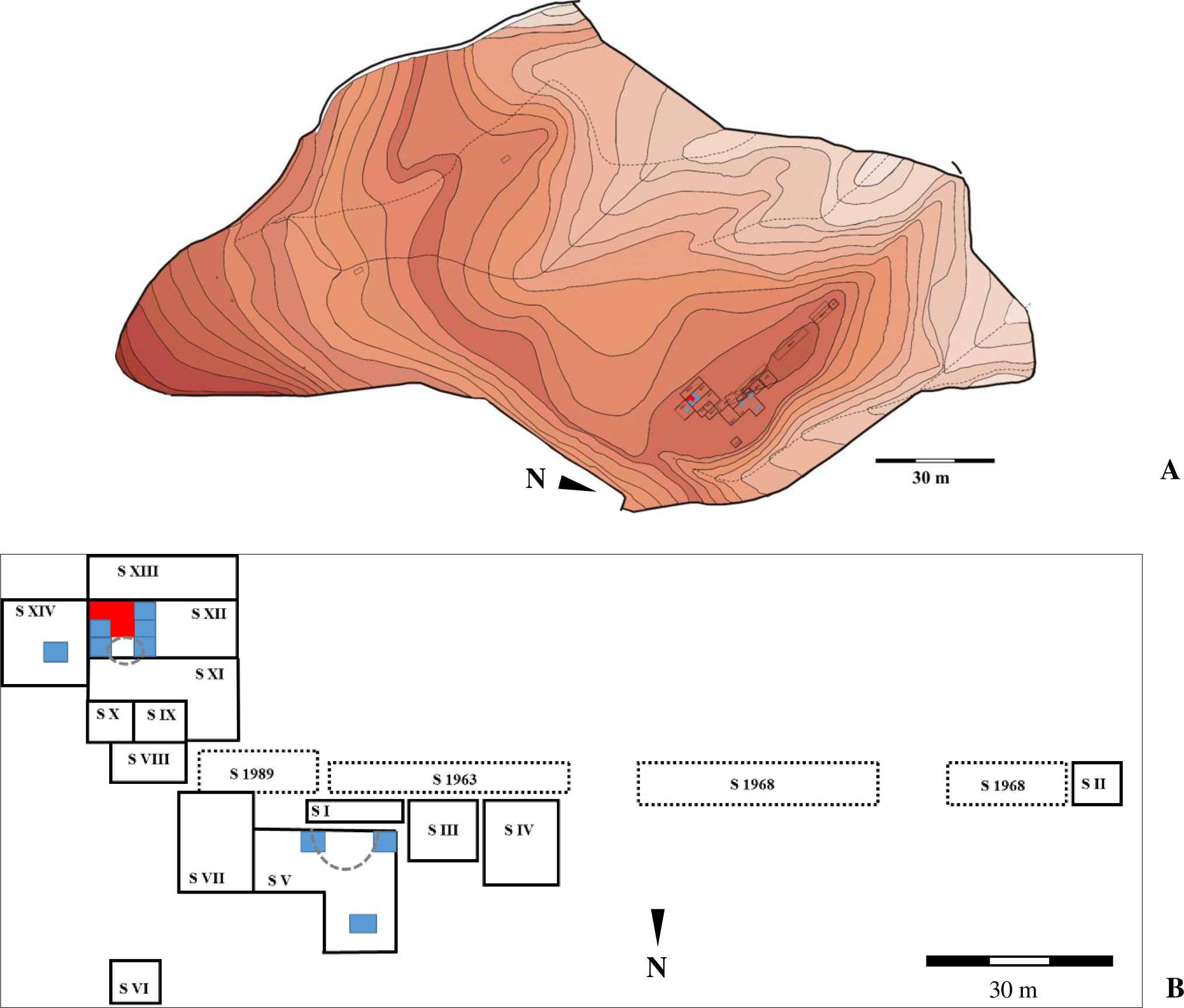

Supplement: S1 Fig — (A) Position of the sections on the topographic map of site. (B) Plan of excavated sections and the position of squares where the perforated shells were found (blue square-Lithoglyphus naticoides and apertus; red square-Homalopoma sanguineum and L. naticoides; the interrupted line indicates the location of hearths). (TIF) [file pone.0214932.s001.tif]

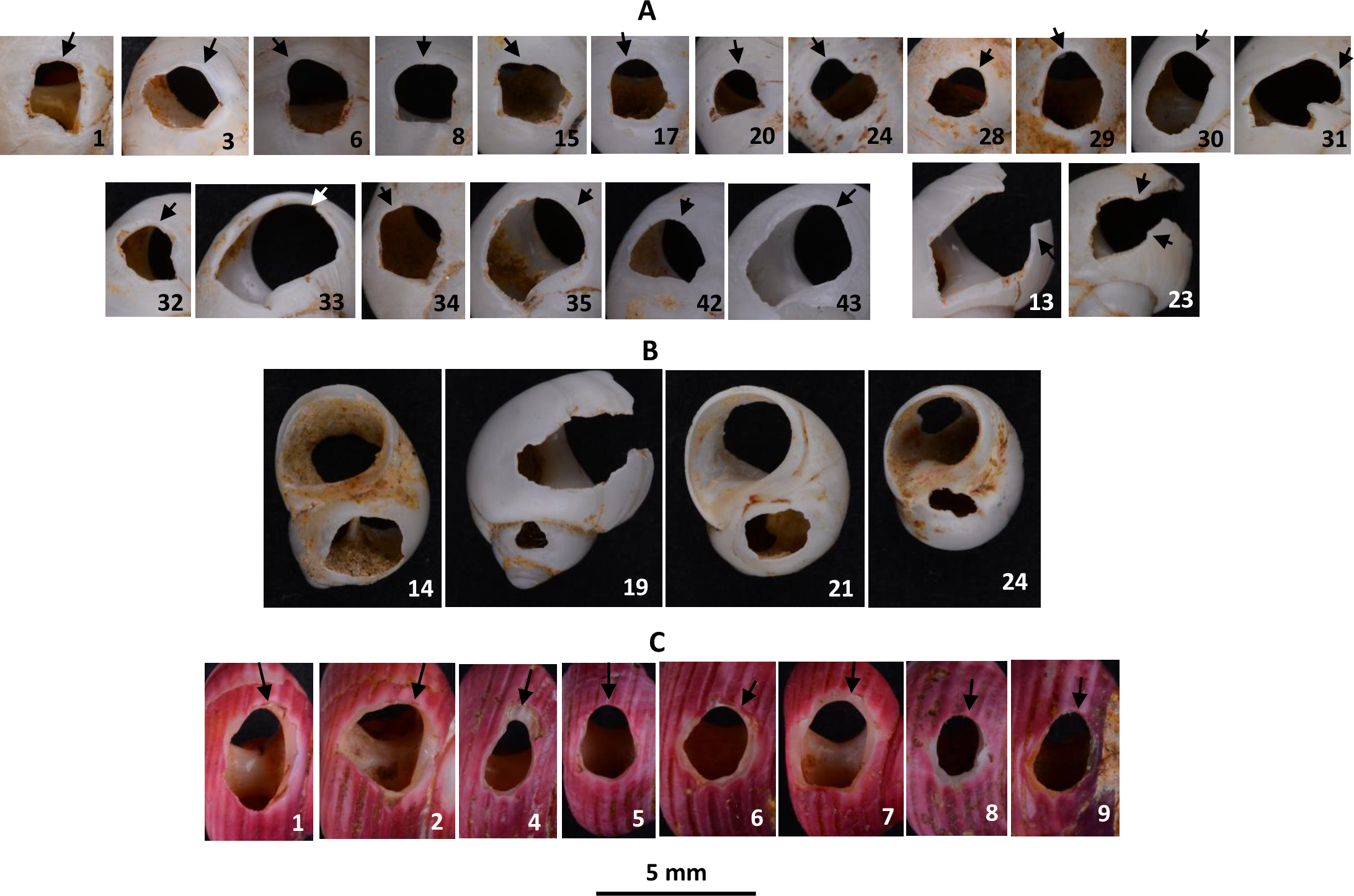

Supplement: S2 Fig — (A) Perforations of the Lithoglyphus naticoides shells. (B) Holes on the surface of Lithoglyphus naticoides shells. (C) Perforations of the Homalopoma sanguineum shells. (TIF) [file pone.0214932.s002.tif]
